# Signal-based spatial domain identification of spatially resolved transcriptomics with multigraph fusion

**DOI:** 10.1093/bib/bbag052

**Published:** 2026-02-11

**Authors:** Yaxiong Ma, Yu Wang, Xiaoke Ma

**Affiliations:** School of Computer Science and Technology, Xidian University, No. 2 South Taibai Road, Xi’an 710071, Shaanxi, China; Key Laboratory of Smart Human-Computer Interaction and Wearable Technology of Shaanxi Province, Xidian University, No. 2 South Taibai Road, Xi’an 710071, Shaanxi, China; School of Computer Science and Technology, Xidian University, No. 2 South Taibai Road, Xi’an 710071, Shaanxi, China; Key Laboratory of Smart Human-Computer Interaction and Wearable Technology of Shaanxi Province, Xidian University, No. 2 South Taibai Road, Xi’an 710071, Shaanxi, China; School of Computer Science and Technology, Xidian University, No. 2 South Taibai Road, Xi’an 710071, Shaanxi, China; Key Laboratory of Smart Human-Computer Interaction and Wearable Technology of Shaanxi Province, Xidian University, No. 2 South Taibai Road, Xi’an 710071, Shaanxi, China

**Keywords:** spatial domain, pathway activity, spatially resolved transcriptomics, graph fusion

## Abstract

Spatially resolved transcriptomics (SRT) measures transcriptomes of cells within intact biological tissues, providing unprecedented opportunities to investigate tissue micro-environments, where spatial domains are modeled as clusters of spatially neighboring cells. Current methods for the identification of spatial domain from SRT mainly rely on expression profiles and spatial coordinates of cells, which ignore intercellular interactions among them, resulting in high sensitivity and low accuracy. To bridge these gaps, we introduce a novel framework, called SiDMGF (Signal-based Domain identification with Multi-Graph Fusion), that integrates gene set-derived signaling and spatial graphs to jointly model biological context, spatial information, and gene expression of cell embedding, thereby dramatically improving accuracy and robustness of performance of algorithms for spatial domain identification. Experimental results demonstrate that SiDMGF consistently outperforms state-of-the-art methods across multiple benchmark datasets and achieves superior domain identification performance on diverse spatial sequence platforms. Furthermore, we demonstrate that the proposed SiDMGF can also be effectively applied to cancer-related tissue samples, accurately delineating micro-environment heterogeneity within tumor slice.

## Introduction

Tissues of multicellular organisms are composed of diverse cell types to execute biological functions through cell–cell interactions. Recent advances in spatially resolved transcriptomics (SRT) technologies, such as 10$\times $ Visium [1], Xenium [2], Stereo-seq [3], and Slide-seq [4], enable the simultaneous measurement of gene expression and spatial locations of cells, providing unprecedented opportunities to investigate the tissue microenvironments within spatial context [5, 6]. Moreover, spatial domains are critical components of tissue microenvironment, which are defined as the contiguous regions composed of spatially adjacent cells with similar expression patterns [7]. Although identifying spatial domains receives great attention, the high levels of noise and dropout events of SRT data hinder the design and application of algorithms [8].

Based on the principles underlying existing algorithms, existing strategies for spatial domain identification can be broadly categorized into two classes: biological experiment and computational approaches, where the former solely relies on pathologists to manually annotate tissue slices based on H&E-stained images [9], whereas the latter utilizes artificial intelligence to automatically annotate SRT data [10, 11]. These algorithms have their unique advantages and disadvantages. For example, the biological experimental approaches are precise and reliable because expert knowledge is incorporated into the annotations process, providing strong biological interpretability. However, these methods are also criticized for their inefficiency because manual annotation imposes an enormous burden on time and finance. To address these limitations, computational approaches based on machine learning provide a promising alternative for biological experiment by reducing financial and time burdens.

In practice, spatial domains of SRT data correspond to the typical unsupervised clustering that aims to obtain continuous regions of histology images with similar gene expression and spatial information of cells. The most intuitive strategy is to directly apply the conventional unsupervised clustering algorithms, such as Leiden [12] implemented in SCANPY [13], to SRT data for the identification of spatial domains by exploiting transcriptomics of cells. However, these algorithms mistakenly identify discontinuous spatial domains because they ignore spatial information of cells, demonstrating that spatial information is critical for modeling structure of spatial domains. To fully leverage spatial information and gene expression of cells, several spatially aware methods are proposed by fusing spatial and expression information with various strategies. For example, stLearn [14] integrates histology images and spatial coordinates of spots to construct cell networks to smooth expression profiles of cells, and then identifies spatial domains by exploiting the topological structure of networks. In addition, graph neural network (GNN)-based approaches [15], such as SEDR [16], SpaGCN [17], STAGATE [18], GraphST [19], DisConST [20], and MAEST [21], concentrate on learning discriminative features of cells by fully exploring power of deep learning for representation. These algorithms significantly improve the performance of spatial domain identification, demonstrating the superiority of deep representations in characterizing complicated structure of spatial domains. However, these algorithms fail to fully address tumor SRT data, because the aggregation mechanism of GNNs for feature learning reduces their ability to capture local heterogeneity of tumors.

To overcome these limitations, Spatial-MGCN [22] employs a multi-view clustering framework to independently construct graphs of cells based on spatial coordinates and expression profiles of cells. Then, it performs feature learning using graph auto-encoder, followed by feature fusion across different networks. The local structure of spatial domains is captured with the attention mechanisms in graph convolutional networks (GCNs), thereby enhancing the discriminability of features for spatial domain identification. However, Spatial-MGCN adopts a post-fusion strategy for spatial and expression features of cells, reducing the compatibility of heterogeneous features of cells. To address this limitation, MNMST [23] simultaneously learns cell features from spatial and expression networks of cells with joint nonnegative matrix factorization, where these networks are projected into a shared latent subspace to improve the consistence of heterogeneous cell features. To further exploit complementary information in SRT data, MuCST [24] integrates histology images with SRT data to delineate spatial domains with contrastive learning, which significantly improves domain identification accuracy, as consistent morphological features provide global guidance for spatial domain organization.

**Figure 1:**
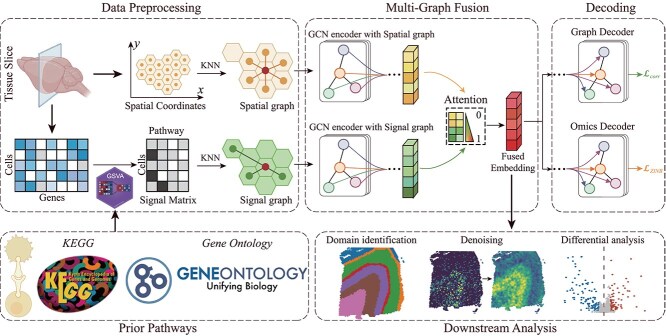
Overview of SiDMGF for identifying domains from SRT data. SiDMGF consists of four components: graph construction, multigraph fusion, decoding, and down-stream analysis. The first procedure employs GSVA to calculate the signal activity matrix of pathways, and then constructs the spatial and signal graph of cells, and the second procedure employs GNN encoders to learn the consistent embedding of cells. The decoding procedure guarantees quality of consistent embedding of cells with dual encoders, and the last procedure testifies the performance of the proposed algorithm.

While considerable success in spatial domain identification is achieved, most existing algorithms focus on learning cell representations from spatial gene expression or histology images, while neglecting key cellular processes and functional relationships among genes at the pathway level, limiting their biological insights for spatial domains. To address this gap, we propose a multigraph fusion framework, called SiDMGF (Signal-based spatial Domain identification with Multi-Graph Fusion), which integrates biological context, spatial information, and gene expression of cells to identify spatial domains from SRT data. Specifically, SiDMGF leverages biological context databases, including KEGG [25] and Gene Ontology [26], and applies the gene set variation analysis (GSVA) algorithm [27] to transform the expression profiles into biological activity profiles. As illustrated in Fig. 1, SiDMGF constructs a spatial graph and a signal graph based on spatial coordinates and activity profiles, respectively. The GNN encoder is then employed to extract graph-specific cell embeddings, and an attention mechanism is adopted to fuse these embedding of cells from various networks [28, 29], where structural constraint is imposed to encourage spatially adjacent cells to share similar embedding. Furthermore, to ensure that the learned cell embeddings comprehensively capture the underlying gene expression, we employ a self-supervised reconstruction loss, which guides the embeddings to preserve expression patterns while being robust to noise and dropout events commonly observed in SRT data [30, 31]. To evaluate the performance of SiDMGF, we benchmark it with nine state-of-the-art algorithms on diverse datasets generated by different SRT technologies. Experimental results demonstrate that SiDMGF consistently achieves the best performance in spatial domain identification, highlighting its potential for analyzing SRT data, and deepening our understanding of architecture and functions of tissues.

## Methods

### Data preprocessing

SiDMGF takes spatial transcriptomics data and gene pathways as inputs, where preprocessing is performed for them. Specifically, for the spatial transcriptomics data, cells (spots) outside of the main tissue region and low-quality cells are removed. The top 3000 highly variable genes are retained for downstream analysis by using Squidpy [32]. The expression profiles of cells are normalized, log-transformed, and scaled according to library size by using SCANPY [13].

In addition, the KEGG pathway database [25] is employed for gene set variation anaysis, which includes 356 and 352 pathways from Homo sapiens and Mus musculus, respectively. GSVA [27] is applied to the preprocessed gene expression profiles, where Poisson kernels are employed to estimate pathway activity levels, thereby constructing the activity strength matrix $AS$ with row $i$ corresponding to the $i$th cell and column $j$ corresponding to the $j$th pathway, where the element $-1 \leq AS_{ij} \leq 1$ denotes the activity strength of pathway $j$ in cell $i$.

### Graph construction

SiDMGF constructs two types of networks: spatial graph of cells $G_{s}$ and signal graph of pathway $G_{a}$, where the former measures proximity of cells in terms of spatial coordinates $S \in \mathbb{R}^{n \times 2}$, and the latter quantifies signal activity strength of cells in terms of matrix $AS \in \mathbb{R}^{n \times k}$ ($n$ and $k$ denote the number of cells and pathways).

For the spatial graph, $G_{s}$ is defined as an unweighted graph, i.e. $G_{s}(i,j)=1$ if and only if its Euclidean distance between the $i$th and $j$th cell is smaller than a predefined threshold $r$ (the selection of $r$ is systematically analyzed in the experimental section). In contrast, the signal graph is constructed as a weighted graph to capture functional relationships among cells. Specifically, a $K$-nearest neighbor algorithm is first applied to determine the graph backbone, after which edge weights are assigned based on the cosine similarity between pathway activity profiles of connected cells. The cosine similarity on edge $(i,j)$ is defined as


(1) \begin{align*}& cos(AS_{i.}, AS_{j.})=\frac{AS_{i.} (AS_{j.})^{^{\prime}}}{|AS_{i.}| |AS_{j.}|},\end{align*}

where $AS_{i.}$ denotes the profile of signal activity vector of the $i$th cell, i.e. corresponding to the $i$th row of matrix $AS$, $(AS_{j.})^{^{\prime}}$ represents the transpose of $AS_{j.}$, and $|AS_{j.}|$ is the length of vector $AS_{j.}$.

### Multigraph fusion

After constructing the spatial and signal graphs, SiDMGF first learns features of cells with two independent GNN encoders, and then fuses these two graphs at the feature level, i.e. learning the consistent features of cells. Specifically, embeddings of cells are first extracted from the spatial graph with graph encoder as


(2) \begin{align*}& H_{s}^{(l+1)} = \sigma \left(\tilde{D}_{s}^{-\frac{1}{2}} \tilde{G}_{s} \tilde{D}_{s}^{-\frac{1}{2}} H_{s}^{(l)} W_{s}^{(l)} \right),\end{align*}

where $\sigma $ denotes the activation function (ReLU by default), $H_{s}^{(l)}$ represents the cell embeddings at the $l$th layer of the GNN encoder, $H_{s}^{(0)}$ corresponds to the input gene expression matrix of cells, $\tilde{G}_{s} = G_{s} + I$ denotes the diagonal degree matrix of spatial graph $\tilde{G}_{s}$, $\tilde{D}_{s}$ is the corresponding diagonal degree matrix, and $W_{s}^{(l)}$ is the trainable weight matrix at the $l$th layer. Analogously, cell embeddings from the signal graph are obtained as


(3) \begin{align*}& H_{a}^{(l+1)} = \sigma \left(\tilde{D}_{a}^{-\frac{1}{2}} \tilde{G}_{a} \tilde{D}_{a}^{-\frac{1}{2}} H_{a}^{(l)} W_{a}^{(l)} \right).\end{align*}

To capture the heterogeneity of tissues, the attention mechanism [28] is adopted to ensure embeddings of cells capture local structure of graphs. Specifically, the attention coefficient $\alpha _{i}^{(a)}$ for the $i$th cell of graph $G_{a}$ is defined as


(4) \begin{align*}& \begin{cases} \alpha_{i}^{(a)} = W_{2}^{(a)} \cdot \tanh \left(W_{1}^{(a)} \mathbf{h}_{i}^{(a)} + \mathbf{b}^{(a)} \right)\\[4pt] \alpha_{i}^{(s)} = W_{2}^{(s)} \cdot \tanh \left(W_{1}^{(s)} \mathbf{h}_{i}^{(s)} + \mathbf{b}^{(s)} \right) \end{cases}\!\!\!\!\!\! ,\end{align*}

where $W_{1}$ and $W_{2}$ denote the trainable weight matrices, and $\mathbf{b}$ denotes the bias term of the GCN. The attention coefficients are further normalized using the softmax function. And, fuses the cell embeddings from the spatial and signal graphs using a multilayer perceptron (MLP), formulated as


(5) \begin{align*}& H = \textrm{MLP}(\alpha^{(s)} H_{s} + \alpha^{(a)} H_{a}),\end{align*}

where $H$ denotes the fused consistent cell embeddings, and $\alpha =\{\alpha _{1},\ldots ,\alpha _{n}\}$ are the attention coefficient vectors of cells.

### Decoding and training

To ensure that the fused cell embeddings consistently capture both the expression and spatial information from graphs [30, 31], SiDMGF adopts two decoders: a graph decoder and an omics decoder, as shown in Fig. 1. Specifically, SiDMGF minimizes the difference between the reconstructed spatial graph $\hat{G}_{s}$ and the original one $G_{s}$ as


\begin{align*} \mathcal{L}_{spatial} =& \sum_{i,j} G_{s}(i,j) \log \big( \phi(\hat{G}_{s}(i,j)) \big) \nonumber \\ &+ (1 - G_{s}(i,j) \log \big( 1 - \phi(\hat{G}_{s}(i,j)) \big), \nonumber \end{align*}

where $\phi (\cdot )$ is the sigmoid activation function.

On the reconstruction of gene expression of cells, SiDMGF adopts the zero-inflated negative binomial (ZINB)-based decoder to address noise and dropout events commonly observed in SRT data. The negative binomial (NB) distribution [33] is widely used to model count-based RNA-seq data to address over-dispersion as


(6) \begin{align*}& NB(x_{i}|\mu_{i}, \theta_{i}) = \frac{\Gamma (x_{i}+\theta_{i})}{x_{i}!\Gamma(\theta_{i})} \left(\frac{\theta_{i}}{\mu_{i}+\theta_{i}}\right)^{\theta_{i}} \left(\frac{\mu_{i}}{\mu_{i} + \theta_{i}}\right)^{x_{i}},\end{align*}

where $\mu _{i}$ is the mean and $\theta _{i}$ is the dispersion parameter. To further account for dropout events, the ZINB distribution extends the NB distribution by introducing an additional dropout parameter $\pi $ as


(7) \begin{align*}& ZINB(x_{i}|\pi, \mu_{i}, \theta_{i}) = (1-\pi) NB(x_{i}|\mu_{i}, \theta_{i}),\end{align*}

where $\pi $, $\mu $, and $\theta $ denote the dropout probability, mean, and dispersion of the ZINB distribution, respectively. The loss function of expression profile can be formulated as the ZINB loss function is formulated as


(8) \begin{align*}& \mathcal{L}_{ZINB} = -\sum_{i=1}^{n} \ln ZINB(x_{i}| \pi, \mu_{i}, \theta_{i}).\end{align*}

The final objective of SiDMGF is formulated as


(9) \begin{align*}& \mathcal{L} = \mathcal{L}_{ZINB} + \lambda \mathcal{L}_{spatial},\end{align*}

where $\lambda $ is hyper-parameter for balancing trade-off between the reconstruction of gene expression reconstruction and spatial graphs.

### Clustering and visualization

SiDMGF performs spatial domain identification based on the learned cell embeddings with Leiden algorithm implemented by SCANPY package [13], and performs differential expression analysis of genes with Wilcoxon rank-sum test. The uniform manifold approximation and projection (UMAP) algorithm [34] is selected to visualize the fused cell embeddings,.

### Benchmarking

We benchmark SiDMGF against seven state-of-the-art algorithms, including the nonspatial method SCANPY [13], as well as spatially aware methods such as SpaGCN [17], stLearn [14], GraphST [19], SEDR [16], Spatial-MGCN [22], MuCST [24], MAEST [21], and DisConST [20]. All methods are executed with the recommended parameter settings to ensure optimal performance. The adjusted Rand index (ARI) is used as the evaluation metric to quantify the agreement between predicted domains and ground truth annotations. The ARI [35] is defined as


\begin{align*}& ARI(P^{*},P)=\frac{\sum_{ij}\binom{n_{ij}}{2}-\left[\sum_{i}\binom{n_{i}}{2}+\sum_{j}\binom{n_{j}}{2}\right]/\binom{n}{2}}{\frac{1}{2}\left[\sum_{i}\binom{n_{i}}{2}+\sum_{j}\binom{n_{j}}{2}\right]-\left[\sum_{i}\binom{n_{i}}{2}+\sum_{j}\binom{n_{j}}{2}\right]/\binom{n}{2}}, \end{align*}

where $P^{*}$ denotes the ground truth partition, $P$ is the predicted clustering, $n_{ij}$ is the number of cells in the intersection between cluster $i$ and true class $j$, and $n$ is the total number of cells. Furthermore, to more comprehensively evaluate the performance of all algorithms, the normalized mutual information [36] and F1-score are also employed as complementary evaluation metrics.

## Results

### Overview of SiDMGF

The ultimate goal of SiDMGF is to effectively integrate biological context of genes and spatial transcriptomics data for the identification of spatial domains, which takes pathways, spatial coordinates, and gene expression profiles of cells as inputs. As illustrated in Fig. 1, SiDMGF consists of four components: graph construction, multigraph fusion, decoding, and downstream analysis, where the first three procedures are devoted to techniques of algorithms, and the last one is for the application of SiDMGF. Specifically, the graph construction procedure performs data preprocessing and constructs the spatial and signal graphs of cells from the input data. The multigraph fusion component integrates these graphs to learn the fused and consistent cell embeddings using GCNs. The decoding component further refines the learned embeddings by reconstructing both spatial structures and pathway signals, ensuring that the fused representations preserve essential information from the original data.

In detail, SiDMGF first employs GSVA to transform raw gene expression of cells into a signal matrix to quantify the activity of pathways associated with each cell, which bridges gene sets (pathways) and cells through expression profiles. By leveraging the pathway activity matrix together with the spatial coordinates of cells, SiDMGF constructs a signal graph and a spatial graph, providing a comprehensive representation of spatial transcriptomics data. On the feature learning issue, SiDMGF employs graph encoders to extract graph-specific cell embeddings by exploiting the topological structures from the constructed graphs. Furthermore, an attention mechanism is introduced to adaptively encourage cell features to capture local structure of networks. To ensure quality of the fused and consistent cell embeddings, the proposed algorithm employs dual encoders to preserve expression and signal graph by minimizing approximation the constructed and original graphs, where graph decoder reconstructs the spatial graph, and omics decoder restores the gene expression profile of cells. Finally, we validate the performance of SiDMGF with downstream analyses, and it consistently outperforms baseline methods, demonstrating its effectiveness for analyzing SRT data, providing alternative for effectively analyzing SRT data with noise and dropout events.

### SiDMGF reveals cortex layers from human DLPFC

**Figure 2:**
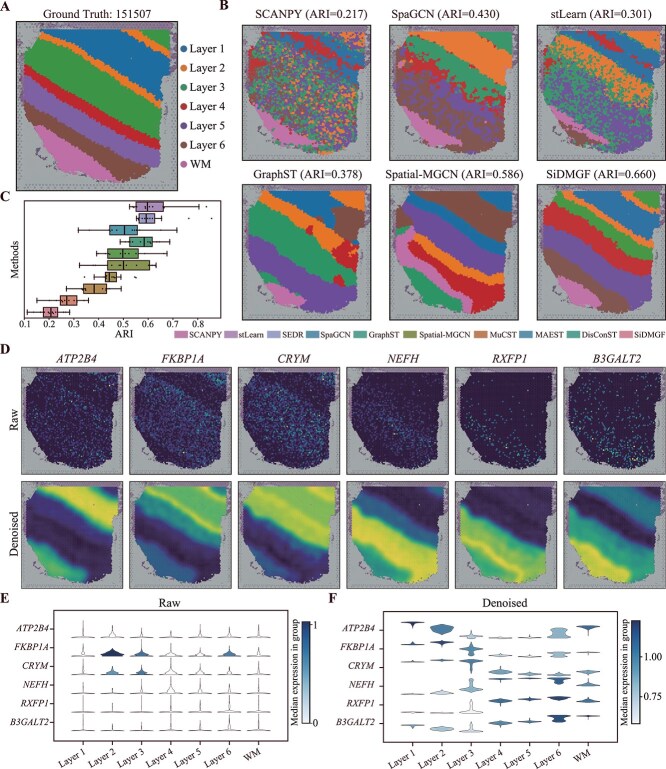
SiDMGF accurately reveals cortex layers from human DLPFC dataset. (A) The manual annotation of six cortical layers and the WM layer in slice 151507 of DLPFC dataset. (B) Visualization of spatial domains identified by nonspatial SCANPY, and spatial-aware SpaGCN, stLearn, GraphST, Spatial-MGCN, and SiDMGF in slice 151507. (C) Boxplot of ARIs of all methods for spatial domains across the 12 slices of DLPFC, where the $x$-axis denotes ARIs of different methods labeled by different colors. The center line, box limits, and whiskers denote the median, upper and lower quartiles, and 1.5$\times $ interquartile range, respectively. (D) Visualizations of the raw spatial expressions (top) and reconstructed slice (bottom) of six layer-marker genes of slice 151507. (E) Violin plots of the raw expression of layer-marker genes. (F) Violin plots of the SiDMGF-denoised expressions of layer-marker genes.

We first evaluate the performance of SiDMGF on the human dorsolateral prefrontal cortex (DLPFC) dataset [37] sequenced using 10$\times $ Visium, which contains 12 slices sequenced from three individuals. Each slice is annotated into six cortical layers (Layer 1–Layer 6) and the white matter (WM) layer with the known marker genes and morphological features. Figure 2A visualizes slice 151507 of DLPFC with the seven layers labeled by different colors, and Fig. 2B illustrates performance of various algorithms for the corresponding slice. Specifically, SiDMGF achieves the best performance for identifying spatial domains with ARI of 0.660, which significantly outperforms the best baseline DisConST with ARI 0.598, achieving $\sim $6.1% improvement. Notice that even though Spatial-MGCN (ARI=0.586), GraphST (ARI=0.378), and SpaGCN (ARI=0.430) successfully delineate major cortical layers, such as Layer 1 and Layer 5, only SiDMGF accurately resolves all seven cortical layers. Particularly, the proposed algorithm precisely distinguishes Layer 2 and Layer 3, whereas all these baselines fail to discriminate these two spatial domains. We further visualize cell embeddings obtained by SiDMGF and baselines with UMAP (Supplementary Fig. S1), where cell embeddings obtained by SiDMGF accurately separates different known cortex layers that the baselines fail to address, supporting the biological relevance of the identified spatial domains.

Then, we apply SiDMGF and baselines to all these 12 slices of DLPFC dataset, and the performance distributions of the algorithms are shown in Fig. 2C and Supplementary Figs S2–S5, where SiDMGF achieves the highest ARI of 0.627 $\pm $ 0.089 (median $\pm $ standard deviation). In comparison, the best baseline DisConST attains an ARI of 0.623 $\pm $ 0.089, while Spatial-MGCN achieves a substantially lower ARI of 0.498 $\pm $ 0.092. These results highlight the importance of incorporating biological context into multigraph fusion for the identification of spatial domains. We also notice that the nonspatial method SCANPY achieves the worst performance with an ARI of 0.208 $\pm $ 0.049, which is significantly lower than all spatially aware methods, further demonstrating the necessity of leveraging spatial information for identifying spatial domains. We further compare the running time and memory usage of SiDMGF with all baseline methods across all DLPFC slices (Supplementary Fig. S6), where SiDMGF is both computationally efficient and scalable, reaches a good trade-off between running time and memory usage, and achieves the best performance on the identification of spatial domains. We also validate the performance of SiDMGF for denoising raw gene expression with biomarker genes. Specifically, we compare the expression level of cortical layer–specific marker genes between the original (upper panel) and reconstructed data (bottom panel) for slice 151507, as shown in Fig. 2D, where each column corresponds to a biomarker gene. For example, *ATP2B4* displays distinct enrichment in Layers 2 and 6, which is consistent with previous studies [38], whereas its raw spatial expression is highly noisy and indistinct. Similar improvements are also observed for other canonical bio-marker genes, including *FKBP1A*, *CRYM*, *NEFH*, *RXFP1*, and *B3GALT2*.

To further assess the effect of reconstruction, we compare the raw (Fig. 2E) and reconstructed (Fig. 2F) expression distributions using violin plots, where the reconstructed expressions reveal markedly enhanced spatial patterns of layer-marker genes, consistently matching the manual annotations of tissue structures. These results demonstrate that the omics decoder effectively captures the global distribution of SRT data and reconstructs gene expression, thereby mitigating noise and dropout events and further validating the robustness of SiDMGF.

### SiDMGF reveals complex layers in mouse brain

**Figure 3:**
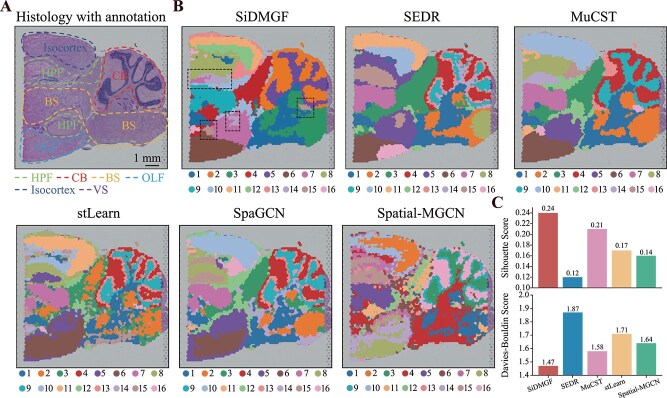
SiDMGF identifies complex spatial domain structures in mouse posterior brain data. (A) Annotated histology image of mouse posterior brain slice. (B) Spatial domains identified by different methods, illustrating their ability to delineate anatomical structures in the posterior brain, where the black dashed lines highlight a narrow region that is correctly delineated by SiDMGF but not captured by most baseline methods. (C) Barplots of SC (top) and Davies–Bouldin (bottom) score of different methods.

We further apply these algorithms to the mouse posterior brain slice sequenced by 10$\times $ Visium, which includes more complex spatial domains. This dataset is annotated with anatomical reference annotations obtained from the Allen Mouse Brain Atlas (Fig. 3A). Figure 3B visualizes the domains obtained by different algorithms, where each spatial domain are labeled with different colors. It is easy to assert that SiDMGF consistently outperforms all these compared methods. Specifically, all baselines successfully identify the cerebellum (CB) region, whereas only SiDMGF, stLearn, and MuCST accurately discover the ventricular system (VS) region (highlighted by dashed boxes in Fig. 3B). Furthermore, SiDMGF successfully identifies the dentate gyrus within the hippocampal formation (HPF), which is consistent with the reference annotations. Only stLearn is able to discover the dentate gyrus in HPF region, while SiDMGF demonstrates strong regional continuity with fewer noise points compared with stLearn.

Since no cell-level annotations are available for the posterior mouse brain dataset, we employ the Silhouette Coefficient (SC) [39] and Davies–Bouldin Index (DB) [40] implemented by Sklearn [41] to quantitatively assess the compactness and separation of spatial domains from perspective of computation, and we find that SiDMGF achieves the highest SC score and the lowest DB score among all methods, indicating that the identified domains are more accurate than baselines from a computational perspective (Fig. 3C), suggesting that SiDMGF is also promising for characterizing complex spatial domains in the mouse brain. Overall, the experimental results demonstrate that SiDMGF improves the identification of spatial domains in SRT data across diverse tissue types.

### SiDMGF precisely reveals cancer-related spatial domains from human breast cancer slice

**Figure 4:**
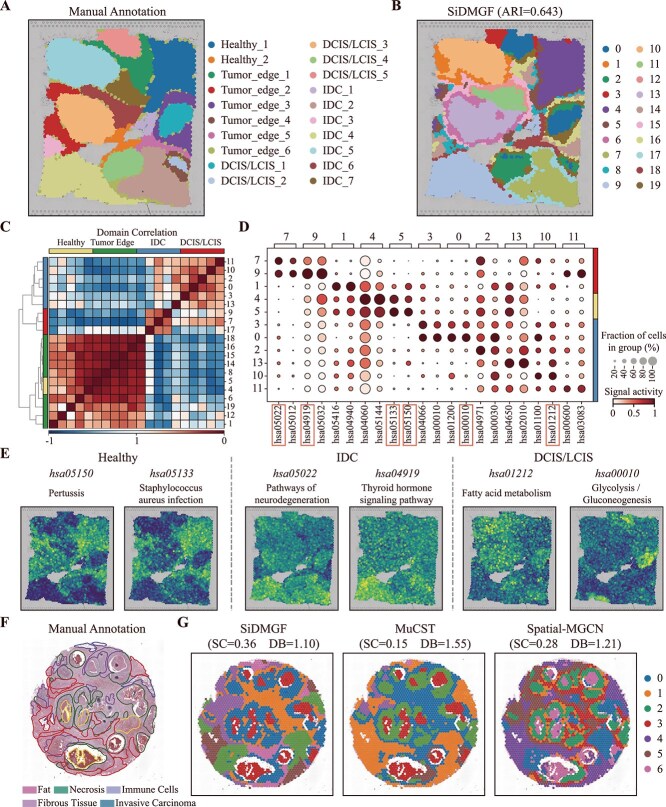
SiDMGF precisely reveals cancer-related spatial domains from human breast cancer slice. (A) Visualization of manual annotation of human breast cancer slice, including Healthy, Tumor edge, DCIS/LCIS, and IDC regions. (B) Spatial domains identified by SiDMGF. (C) Heatmap of Pearson correlation coefficients among the identified domains. Morphotype annotations on the left are obtained by mapping the identified domains to the manual annotations. (D) Dotplot of domain-specific pathway activities, where color intensity represents the median pathway activity within each domain, and dot size indicates the proportion of cells expressing the pathway. (E) Visualization of signal activity strength of pathways for Healthy, IDC, and DCIS/LCIS. (F) Manual annotation of human breast cancer sample DCIS. (G) The spatial domains identified by SiDMGF, MuCST, and Spatial-MGCN.

To further evaluate performance of SiDMGF, the human breast cancer slice sequenced using 10$\times $ Visium with 36 601 genes and 3798 cells is selected, which is manually annotated by pathologists [16]. The 20 regions (spatial domians) are grouped into four major morphotypes: ductal carcinoma *in situ*/lobular carcinoma *in situ* (DCIS/LCIS), healthy tissue (Healthy), invasive ductal carcinoma (IDC), and tumor edge regions (Fig. 4A). Figure 4B visualizes the spatial domains identified by SiDMGF, achieving an ARI of 0.643, which is substantially higher than those of the baseline methods (Supplementary Fig. S7). In particular, SiDMGF accurately delineates cancer-related domains that are highly consistent with the manual annotations. In contrast, the domains identified by the baseline methods exhibit lower spatial continuity (Supplementary Fig. S7). These results demonstrate that SiDMGF is also effective for characterizing cancer-related spatial domains. Moreover, SiDMGF successfully distinguished tumor edge regions (e.g. domains 1, 12, and 19) from adjacent tumor and healthy regions, whereas baselines fail to make such distinctions. The identification of transition zones from healthy to tumor domains highlights that incorporating pathway-level signal activities facilitates the identification of spatial domains.

Tumor heterogeneity is critical for cancer diagnosis and therapy [42], and we hypothesize that tumor heterogeneity can also be reflected by spatial domains. The heatmap of the correlation matrix among these spatial domains identified by SiDMGF is visualized in Fig. 4C, where the correlation coefficients are calculated using the Pearson correlation of median pathway activities across cells within each domain, and it reveals that SiDMGF separates domains into two distinct groups, where the lower left block are mainly composed of Healthy and Tumor edge regions, consistent with their spatial colocalization, as healthy domains are clustered together and are surrounded by Tumor edge regions. In contrast, the upper right block predominantly consists of cancer-related domains, including DCIS/LCIS and IDC regions. The correlation patterns further indicate that tumor edge regions serve as transitional states between the healthy and malignant regions, consistent with the expert knowledge of breast cancer [43]. These results prove that pathway activities within spatial domains also serve as biomarkers to characterize tumor heterogeneity.

We further perform the differential pathway activity analysis across the three major morphotypes, Healthy, IDC, and DCIS/LCIS domains, to identify domain-specific pathways (Fig. 4D). Figure 4E visualizes the spatial distribution of pathways. Notably, the two immune-related pathways: *hsa05150* [44] and *hsa05133* [45] are significantly enriched in Healthy regions, suggesting that these two pathways involve core components of inflammatory response, indicating a robust immune surveillance niche in healthy tissues.

Also, disease-related signaling pathways, such as *hsa05022* [46] and *hsa04919* [47], are enriched in domains 5 and 3 of IDC. For DCIS/LCIS, energy production and metabolism pathways, such as *hsa01212* [48] and *hsa00010* [49], are well-known hallmarks of cancers; their domain-specific enrichment suggests that spatial metabolic reprogramming is a key driver of tumor progression and contributes to the distinct functional heterogeneity observed across different malignant stages. Interestingly, the spatial distributions of *hsa01212* and *hsa00010* are different, suggesting further sub-stratification within the DCIS/LCIS subtype. Interestingly, these findings repeat in the additional breast cancer slice with 17 943 genes across 2518 cells, which is manually segmented by pathologists based on histology images into five domains, including fat, fibrous tissue, immune cells, invasive carcinoma, and necrosis (Fig. 4F). Domains identified by SiDMGF are more spatially coherent than those generated by baselines, which are most consistent with the manual annotations. As cell-level annotations are not available for this dataset, we again employ the SC and DB to quantitatively evaluate performance of spatial domains. As demonstrated in Fig. 4G, SiDMGF achieves the highest SC and the lowest DB scores.

Overall, these results indicate that the leverage of pathway activity strength facilitates the accurate identification of cancer-related spatial domains, further demonstrating the robustness and advantages of SiDMGF for spatial domain identification.

### SiDMGF is applicable to datasets generated with different platforms

**Figure 5:**
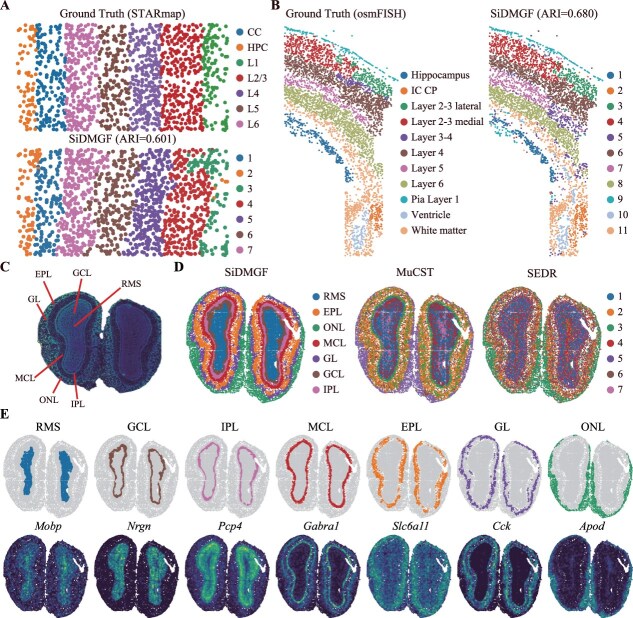
SiDMGF is applicable for spatially resolved data sequenced various platforms with different resolutions and genome coverages.(A) The manual annotation of mouse visual cortex sequenced by STARmap (top), and spatial domains identified by SiDMGF (bottom). (B) The manual annotations of mouse cortex dataset generated by osmFISH (left), and spatial domains identified by SiDMGF (right). (C) DAPI-stained image of mouse olfactory bulb dataset generated by Stereo-seq platform. (D) The spatial domains identified by SiDMGF (left), MuCST (middle), and SEDR (right). (E) Visualization of spatial domains identified by SiDMGF (top), and the spatial expression patterns of known markers.

In addition to 10$\ ^{\ast} $ Genomic Visium data, we further apply these algorithms with various SRT datasets generated by different platforms with various spatial resolutions and gene coverage to evaluate its robustness of algorithms. We first evaluate the performance of these methods on the mouse visual cortex dataset generated by STARmap [50] with 1020 genes and 1207 cells, which consists of seven manually annotated cortical layers (Fig. 5A top). SiDMGF outperforms all baselines with an ARI of 0.601 (Fig. 5 bottom, Supplementary Fig. S8A), whereas the best basline, GraphST, achieves an ARI of 0.558, further indicating the benefit of incorporating signal activities for spatial domain identification. Notably, SiDMGF successfully distinguishes the L1 and L2/3 layers, whereas all baselines fail to discriminate them. To investigate the reason for why SiDMGF distinguishes these two domains, we visualize and compare embedding of cells obtained by various methods with UMAP (Supplementary Fig. S8B). It is evident that cell embeddings extracted by SiDMGF clearly separates L1 from L2/3, while these two layers remain mixed by baselines.

We then evaluate SiDMGF on the mouse cortex dataset generated by osmFISH [51], which profiles only 33 genes across 4839 cells, and manually segmented into 11 anatomical domains (Fig. 5B, left panel). Interestingly, SiDMGF substantially outperforms baselines by achieving an ARI of 0.680, whereas the best baseline MuCST with an ARI of 0.507, achieving about 17.3% improvement of performance (Fig. 5B, right panel, Supplementary Fig. S9). Notably, SiDMGF effectively distinguishes the lateral and medial subdivisions of Layer 2/3, where all baselines fail to resolve.

We further evaluate the performance of SiDMGF on datasets with higher spatial resolution and whole-transcriptome coverage, i.e. Stereo-seq dataset from mouse olfactory bulb tissues [16] with subcellular spatial resolution, which measures 27 106 genes in 19 109 cells. The slice is segmented into seven layers based on DAPI-stained image: the granule cell layer (GCL), mitral cell layer (MCL), external plexiform layer (EPL), glomerular layer (GL), rostral migratory stream (RMS), internal plexiform layer (IPL), and olfactory nerve layer (ONL) (Fig. 5C). The spatial domains identified by SiDMGF are consistent with the annotations (Fig. 5D). In contrast, these domains identified by MuCST and SEDR are with more outliers, failing to recover narrow domains. Since no cell-level ground-truth annotation is available for this dataset, we further validate the identified domains by comparing them with the spatial expression patterns of known domain-specific marker genes. Specifically, *Mobp* is adopted for RMS, *Nrgn* for GCL, *Pcp4* for IPL, *Gabra1* for MCL, *Slc6a11* for EPL, *Cck* for GL, and *Apod* for ONL. The observed expression patterns of these markers show that strong association with the domains identified by SiDMGF is observed, which are consistent with previous findings [52–56] (Fig. 5E).

Overall, these results demonstrate that SiDMGF is robust and effective across SRT datasets generated from diverse platforms with various spatial resolutions and gene coverage.

### The ablation study of SiDMGF

To systematically investigate the contribution of each component of SiDMGF, we conduct an ablation study on the DLPFC dataset. We sequentially remove the signal graph, spatial graph, graph decoder, omics decoder, and attention mechanism to assess their individual effect on performance of the proposed algorithm. The variants of SiDMGF are defined as

- **SiDMGF-w/o-signal**: removes the signal graph and corresponding GNN encoder, relying only on the spatial graph for cell embeddings and domain identification.
- **SiDMGF-w/o-spatial**: removes the spatial graph and corresponding GNN encoder, relying only on the signal graph.
- **SiDMGF-w/o-GD**: removes the graph decoder and retains only the omics decoder.
- **SiDMGF-w/o-OD**: removes the omics decoder and retains only the graph decoder.
- **SiDMGF-w/o-AT**: removes the attention layer and assigns equal weights to embeddings from the spatial and signal graphs.

**Figure 6:**
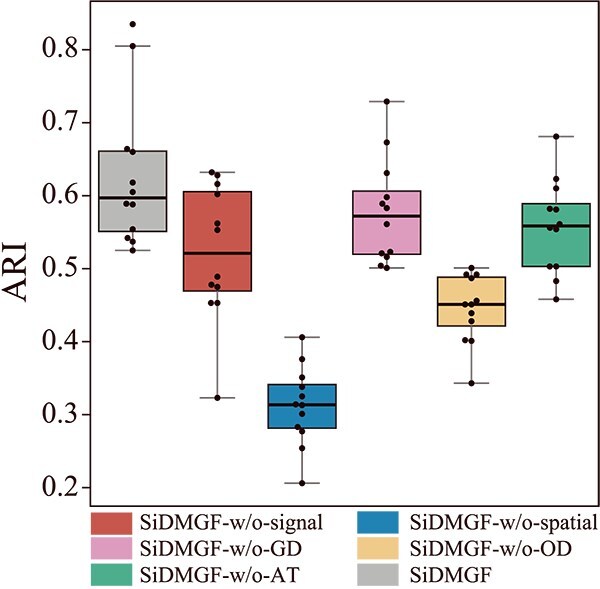
The boxplot of ARI scores of SiDMGF and its variants on the DLPFC dataset.

As demonstrated in Fig. 6, SiDMGF (0.597 $\pm $ 0.097) consistently outperforms all of its variants, demonstrating that each component of the proposed algorithm is necessary. For example, removing the spatial graph results in dramatic reduction of performance, i.e. ARI of SiDMGF-w/o-spatial is 0.314 $\pm $ 0.052, which proves the critical role of spatial information for the identification of spatial domains, and removing signal graph also reduces performance (SiDMGF-w/o-signal, 0.521 $\pm $ 0.089), confirming that the incorporation of pathway activity is also critical for spatial domains. We further investigate contribution of dual decoders. Removing graph decoder (SiDMGF-w/o-GD) results in a slight decrease of performance (0.572 $\pm $ 0.069), suggesting that the structural constraint imposed by the graph decoder enhances continuity and consistency of spatial domains. By contrast, removing the omics decoder (SiDMGF-w/o-OD) results in a severe performance decrease (0.451 $\pm $ 0.047), indicating that the omics decoder plays an essential role in guiding embedding of cells. Finally, removing the attention mechanism (SiDMGF-w/o-AT) results in moderate results (0.559 $\pm $ 0.061), suggesting that in the absence of attention layer, SiDMGF struggles to effectively integrate embedding of cells from different graphs. Furthermore, a comprehensive sensitivity analysis of the two key parameters involved in SiDMGF, $r$ and $\lambda $, demonstrates that the SiDMGF maintains a robust performance across a wide range of parameter settings, and these results also validate the effectiveness of the spatial radius selection strategy adapted from Squidpy, confirming that such approach consistently captures biologically relevant spatial dependencies without requiring extensive hyperparameter tuning (Supplementary Fig. S10).

Overall, the ablation study highlights the importance of jointly integrating pathway activity strength and spatial information for the identification of spatial domains. The combination of decoders and the attention mechanism further ensures quality of embedding of cells, thereby accounting for the superiority of SiDMGF.

## Discussion

In this study, we present a multigraph fusion framework SiDMGF that integrates pathway activity strength with spatial and expression information for spatial domain identification from SRT data.

To evaluate performance of SiDMGF, we perform experiments on various datasets generated from different platforms with various resolutions and genome coverages. Experimental results demonstrate that SiDMGF consistently outperforms state-of-the-art baselines in both normal and tumor tissues. Specifically, SiDMGF achieves the best performance on all 12 slices of human DLPFC dataset, which precisely identifies cortical layers with the best ARI values compared with all baselines. SiDMGF also precisely identifies spatial domains with intricate patterns in a more complex mouse posterior brain slice.

Furthermore, SiDMGF achieves the best performance on the human breast cancer dataset, which successfully identifies heterogeneous cancer subtypes such as DCIS/LCIS and IDC, as well as tumor edge regions that all baselines fail to distinguish. The incorporation of pathway-based signal activities further enables the characterization of tumor heterogeneity, revealing domain-specific biological processes and supporting the potential use of pathway activity profiles as biomarkers for clinical applications. We also demonstrate the generalization of SiDMGF across datasets with different spatial resolutions and platforms, including STARmap, osmFISH, and Stereo-seq. Despite challenges such as limited gene panels or subcellular resolution, SiDMGF consistently outperforms baselines, indicating that SiDMGF is broadly applicable to diverse SRT technologies, underscoring its potential as a versatile tool for both biological discovery and clinical research.

Overall, SiDMGF addresses fundamental limitations of current spatial domain identification methods by integrating multilevel biological signals into a unified framework. By simultaneously leveraging gene expression, spatial proximity, and pathway activity strength of cells, SiDMGF not only improves domain identification performance, but also enhances biological interpretability.

**Key Points**

- In this study, we propose a novel multigraph fusion framework SiDMGF, which integrates spatial proximity, pathway activity, and gene expression for spatial domain identification.
- By incorporating pathway activity strength, SiDMGF learns biologically interpretable and discriminative embedding of cells. Moreover, SiDMGF adopts dual decoders to reconstruct gene expression profiles, enhancing the spatial expression patterns of domain-specific marker genes, which addresses noise and high dropout events of SRT data.
- Experimental results demonstrate that SiDMGF consistently outperforms state-of-the-art baselines across various datasets sequenced from different platforms with different spatial resolutions and genome coverages, indicating that SiDMGF is a generalized and promising tool for SRT data.

## Supplementary Material

SiDMGF-Supplementary_File_bbag052

## Data Availability

All datasets employed in this paper are published datasets and are available for download. Specifically, (1) the 12 slices of human DLPFC dataset are accessible from SpatialLIBD at http://spatial.libd.org/spatialLIBD. (2) The mouse posterior brain slice is obtained from 10$\times $ Visium and available at https://www.10xgenomics.com/resources/datasets. (3) The human breast cancer and DCIS slices are sequenced by 10$\times $ Visium and are available at https://www.10xgenomics.com/resources/datasets. (4) The mouse visual cortex slice is sequenced by STARmap and is available at https://figshare.com/articles/dataset/STARmap_datasets/22565209. (5) The mouse cortex somatosensory slice is sequenced by osmFISH and is available at http://linnarssonlab.org/osmFISH/availability. (6) The mouse olfactory bulb slice is sequenced by Stereo-seq and is available at https://github.com/JinmiaoChenLab/SEDR_analyses. The source code and detailed tutorials for SiDMGF are available at https://github.com/xkmaxidian/SIDMGF.
