# Supplementary material for: Signal-based spatial domain identification of spatially resolved transcriptomics with multigraph fusion
Source: Brief Bioinform. 2026 Feb 11;27(1):bbag052. doi: 10.1093/bib/bbag052 (PMC12893220; doi:10.1093/bib/bbag052)
Supplement: SiDMGF-Supplementary_File_bbag052 [file sidmgf-supplementary_file_bbag052.docx]

Supplementary Materials for

**Signal-based domain identification from spatially resolved transcriptomics with multi-graph fusion**

Yaxiong Ma ^1,2^, Yu Wang ^1,2*^, and Xiaoke Ma ^1,2∗^

^1^ School of Computer Science and Technology, Xidian University, No.2 South Taibai Road, Xi'an, 710071, Shaanxi, China.

^2^ Key Laboratory of Smart Human-Computer Interaction and Wearable Technology of Shaanxi Province, Xidian University, No.2 South Taibai Road, Xi'an, 710071, Shaanxi, China.

Corresponding author. Email: [yuwangxdu@163.com](mailto:yuwangxdu@163.com), [xkma@xidian.edu.cn](mailto:xkma@xidian.edu.cn)

**Keywords:** Spatial domain, Pathway activity, Spatially resolved transcriptomics, Graph fusion

**1.Supplementary Figures**


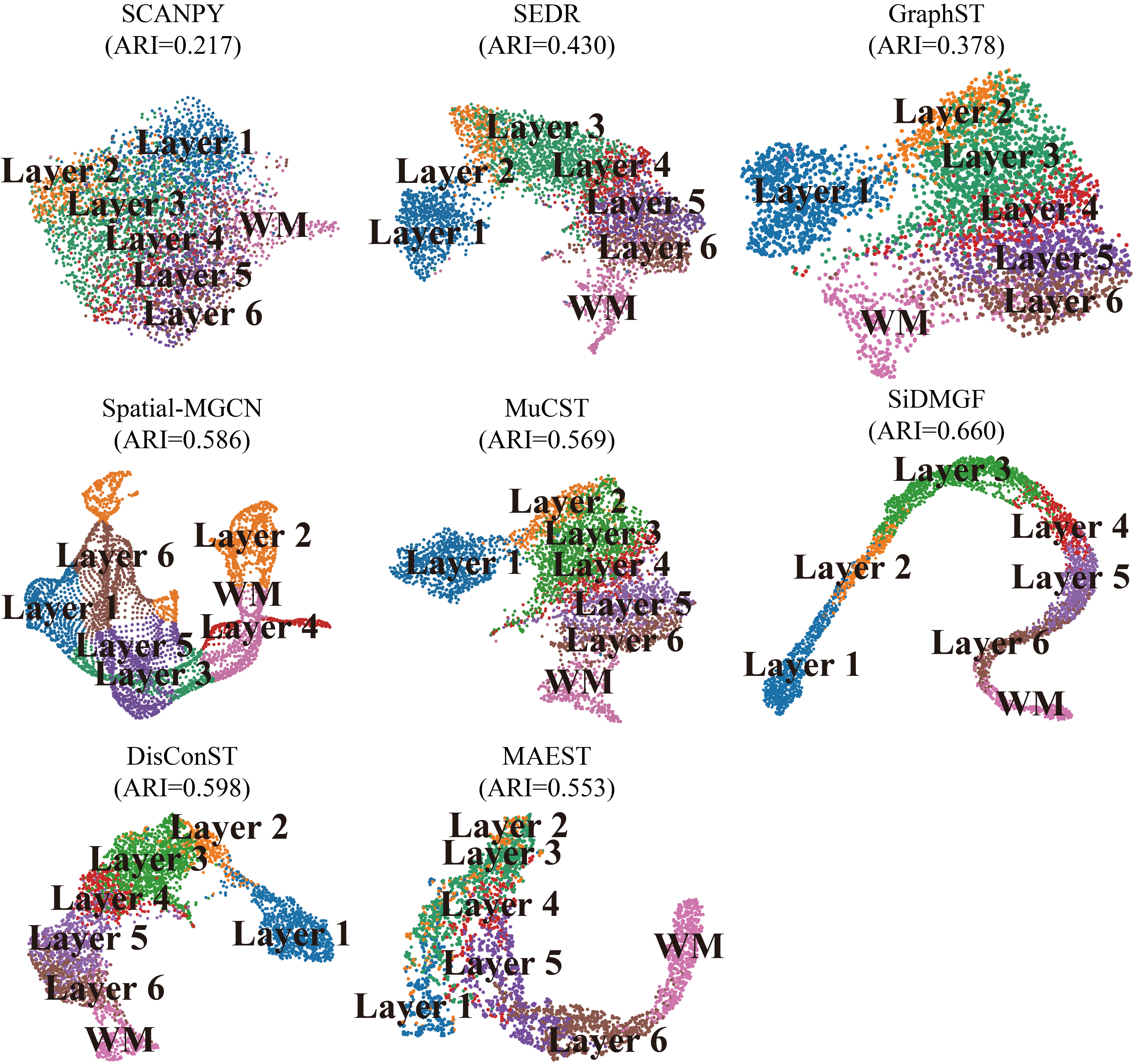


**Fig. S1** UMAP plots of cell embeddings learned by different spatial domain identification methods, where cells are colored by ground truth labels.


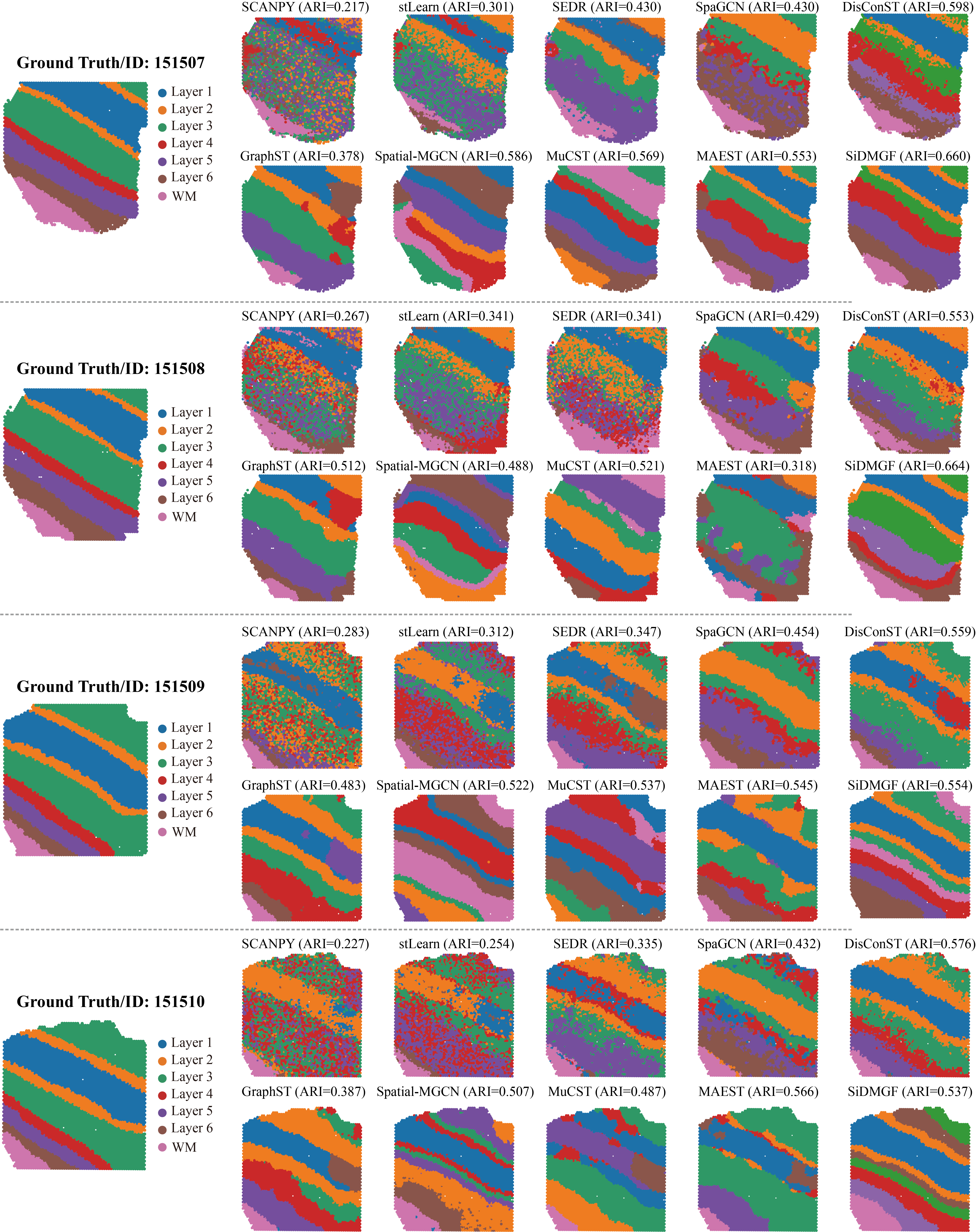


**Fig. S2** Performance of various algorithms for spatial domain identification on Annotated dorsolateral prefrontal cortex (DLPFC, http://spatial.libd.org/spatialLIBD) data (151507, 151508, 151509, 151510), where ground truth spots are mapped on their spatial location, divided into various cortical layers (L1-L6 or L3-L6) and white matter (WM) layer.


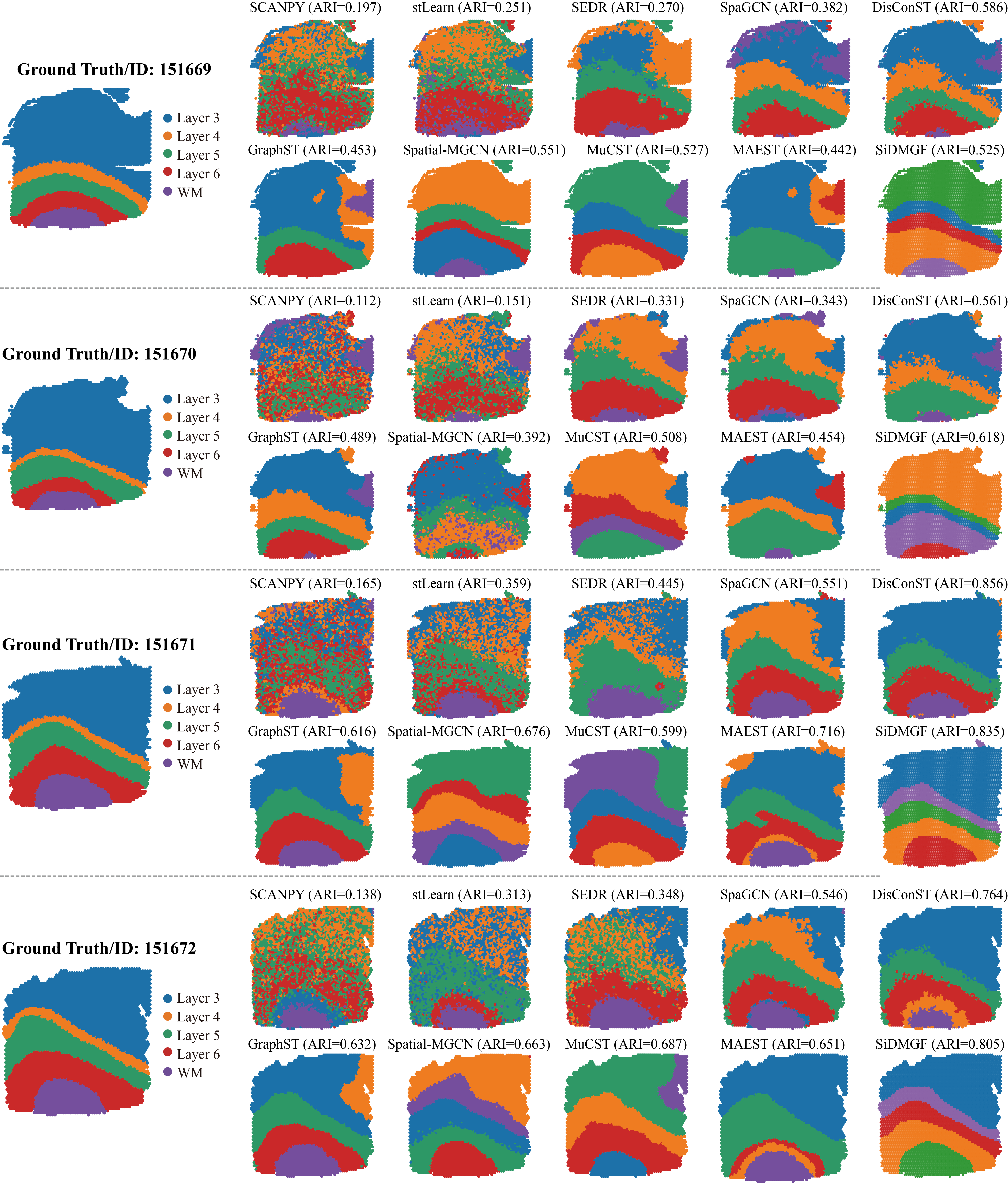


**Fig. S3** Performance of various algorithms for spatial domain identification on Annotated dorsolateral prefrontal cortex (DLPFC, http://spatial.libd.org/spatialLIBD) data (151669, 151670, 151671, 151672), where ground truth spots are mapped on their spatial location, divided into various cortical layers (L1-L6 or L3-L6) and white matter (WM) layer.


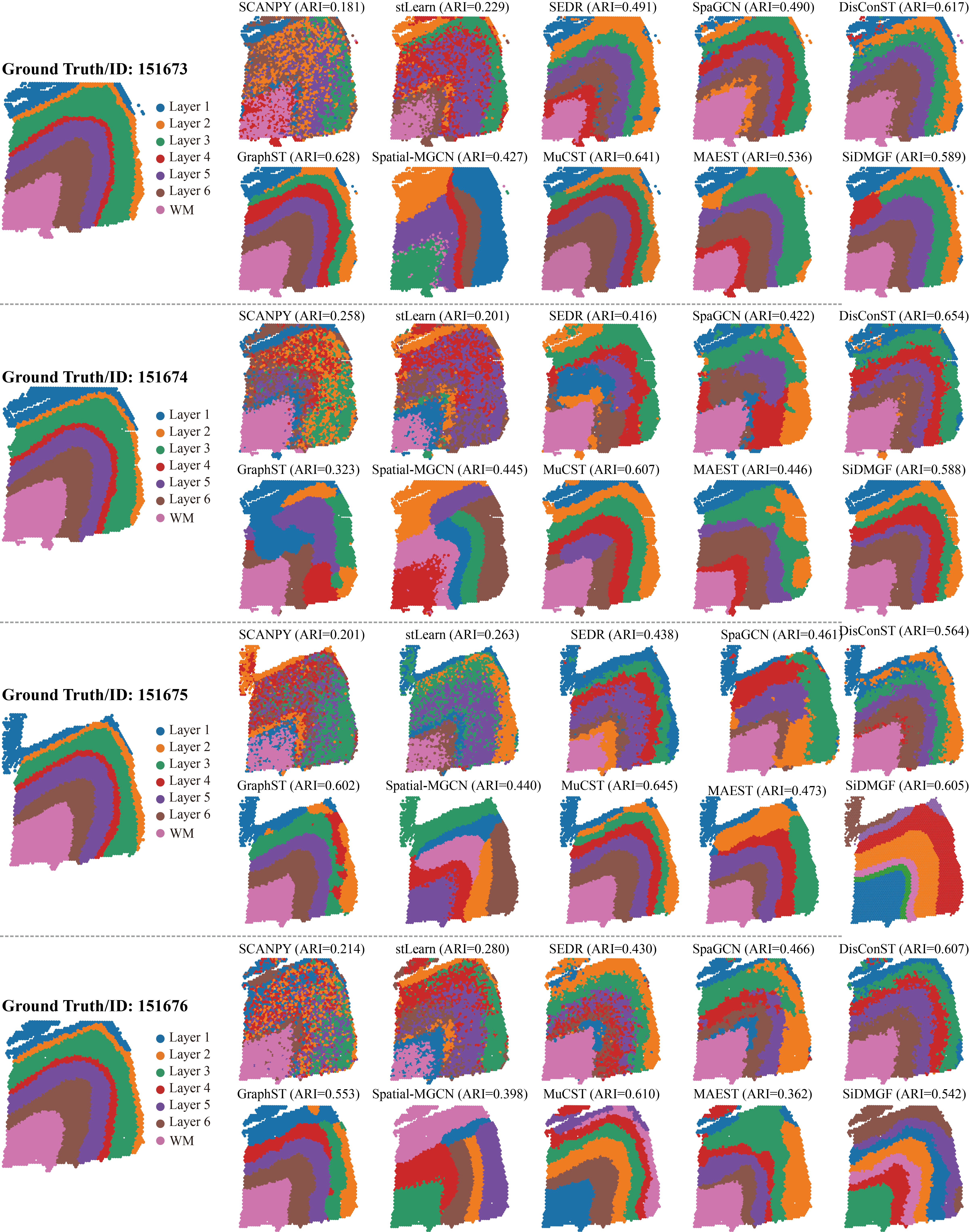


**Fig. S4** Performance of various algorithms for spatial domain identification on Annotated dorsolateral prefrontal cortex (DLPFC, http://spatial.libd.org/spatialLIBD) data (151673, 151674, 151675, 151676), where ground truth spots are mapped on their spatial location, divided into various cortical layers (L1-L6 or L3-L6) and white matter (WM) layer.


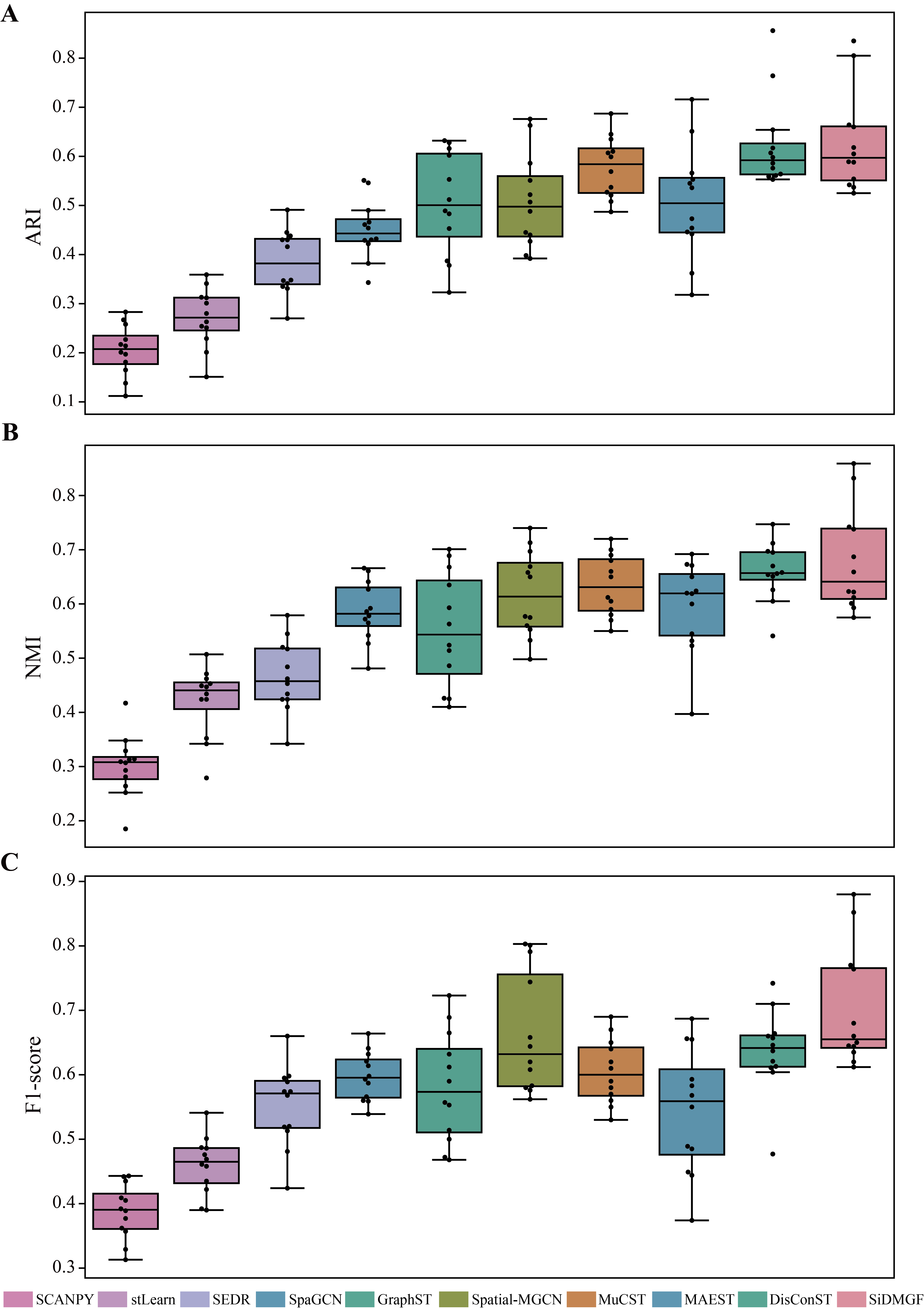


**Fig. S5** Boxplot of accuracy of different algorithms for identifying spatial domains in all 12 slices of DLPFC dataset in terms of various measurements: **(A)** ARI, **(B)** NMI, and **(C)** F1-score, where the center line, box limits and whiskers denote the median, upper and lower quartiles and 1.5$\times$ interquartile range, respectively.


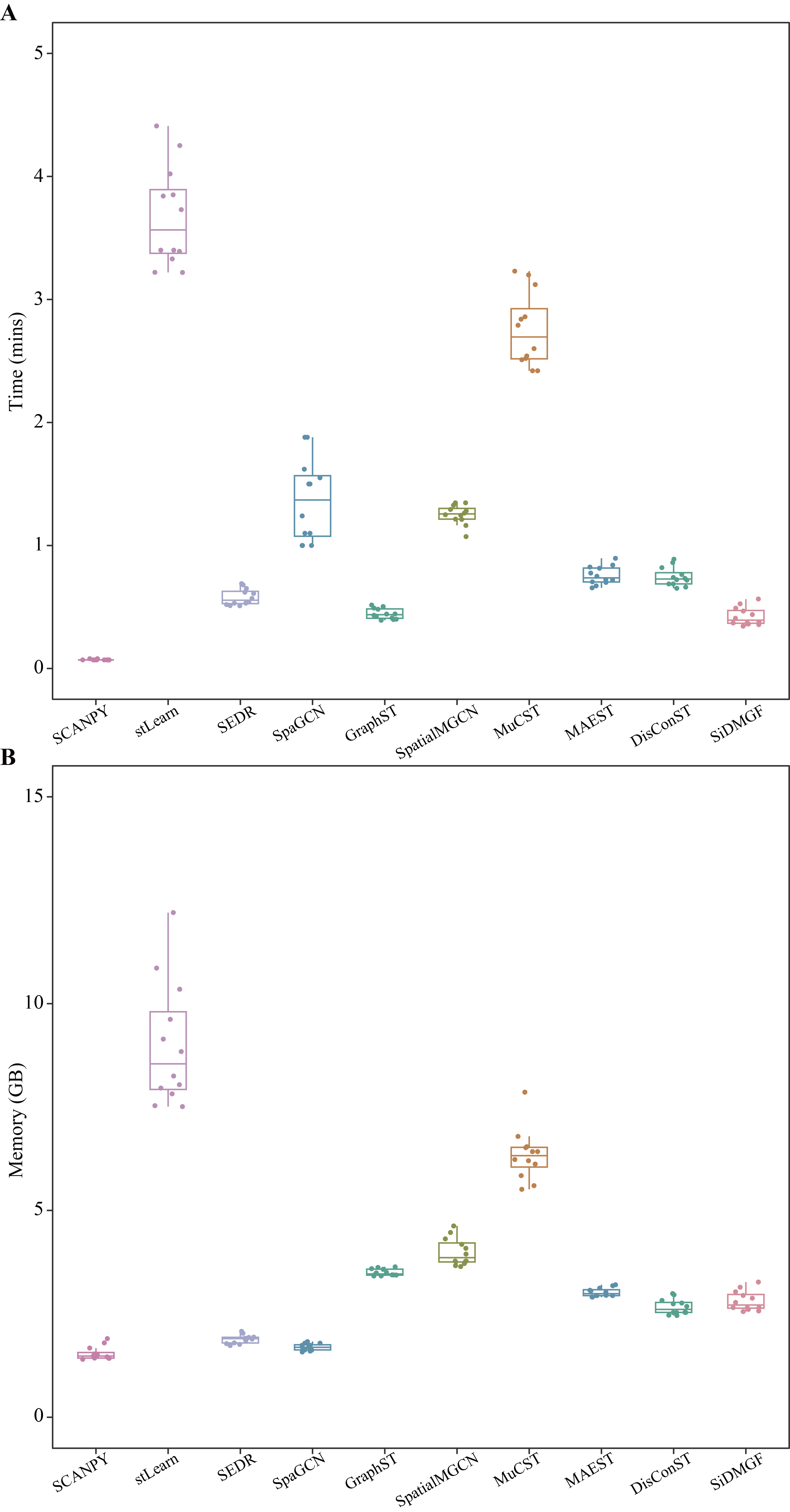


**Fig. S6** Boxplot of running time **(A)** and memory usage **(B)** of different algorithms on all slices of DLPFC dataset, where the center line, box limits and whiskers denote the median, upper and lower quartiles and 1.5$\times$ interquartile range, respectively.


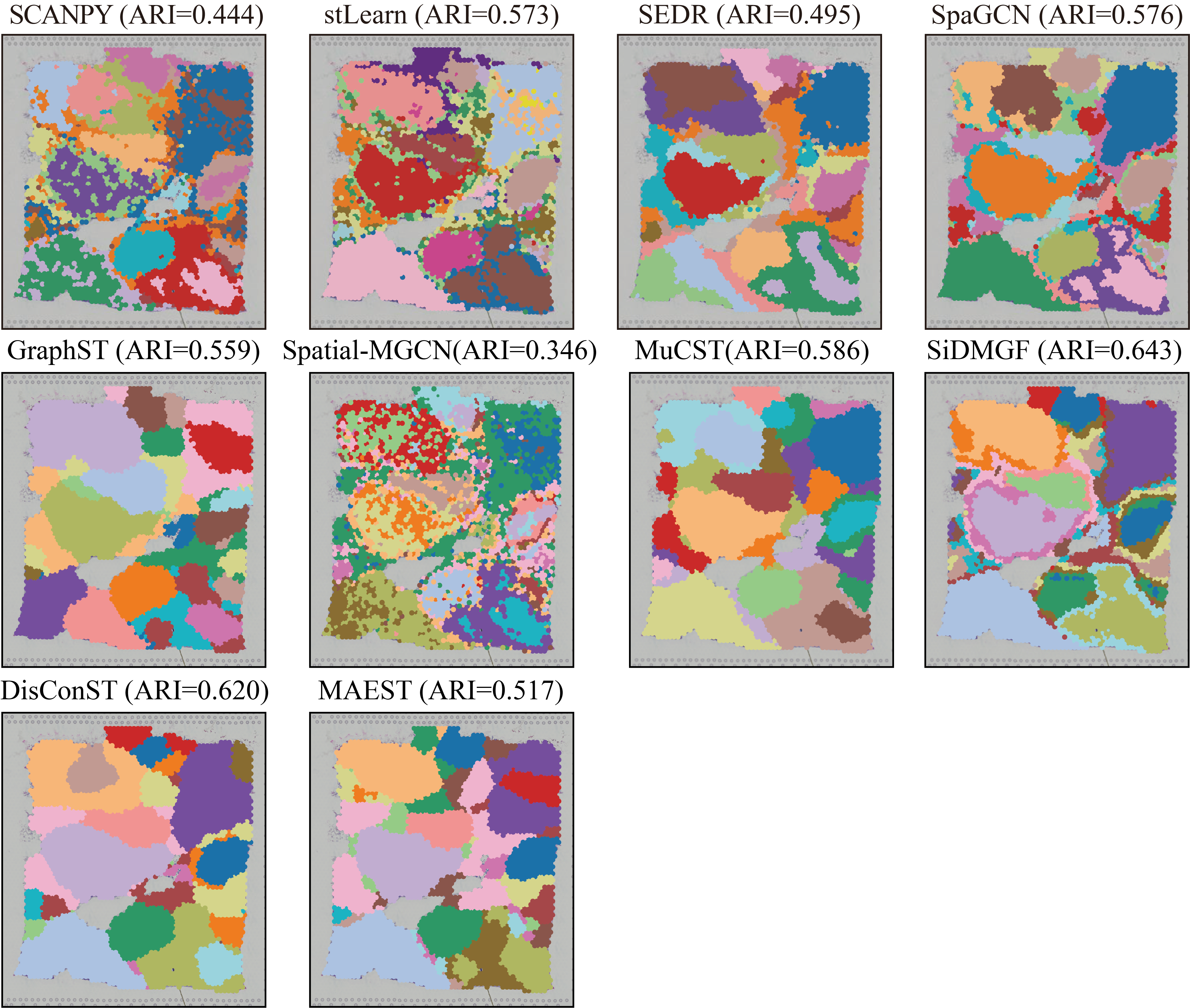


**Fig. S7** Comparison of spatial domains identified by SCANPY, stLearn, SEDR, SpaGCN, GraphST, Spatial-MGCN, MuCST, and SiDMGF on the human breast cancer data, and visualization of identified spatial domains (domains=20), where spots are colored by the identified spatial domains.


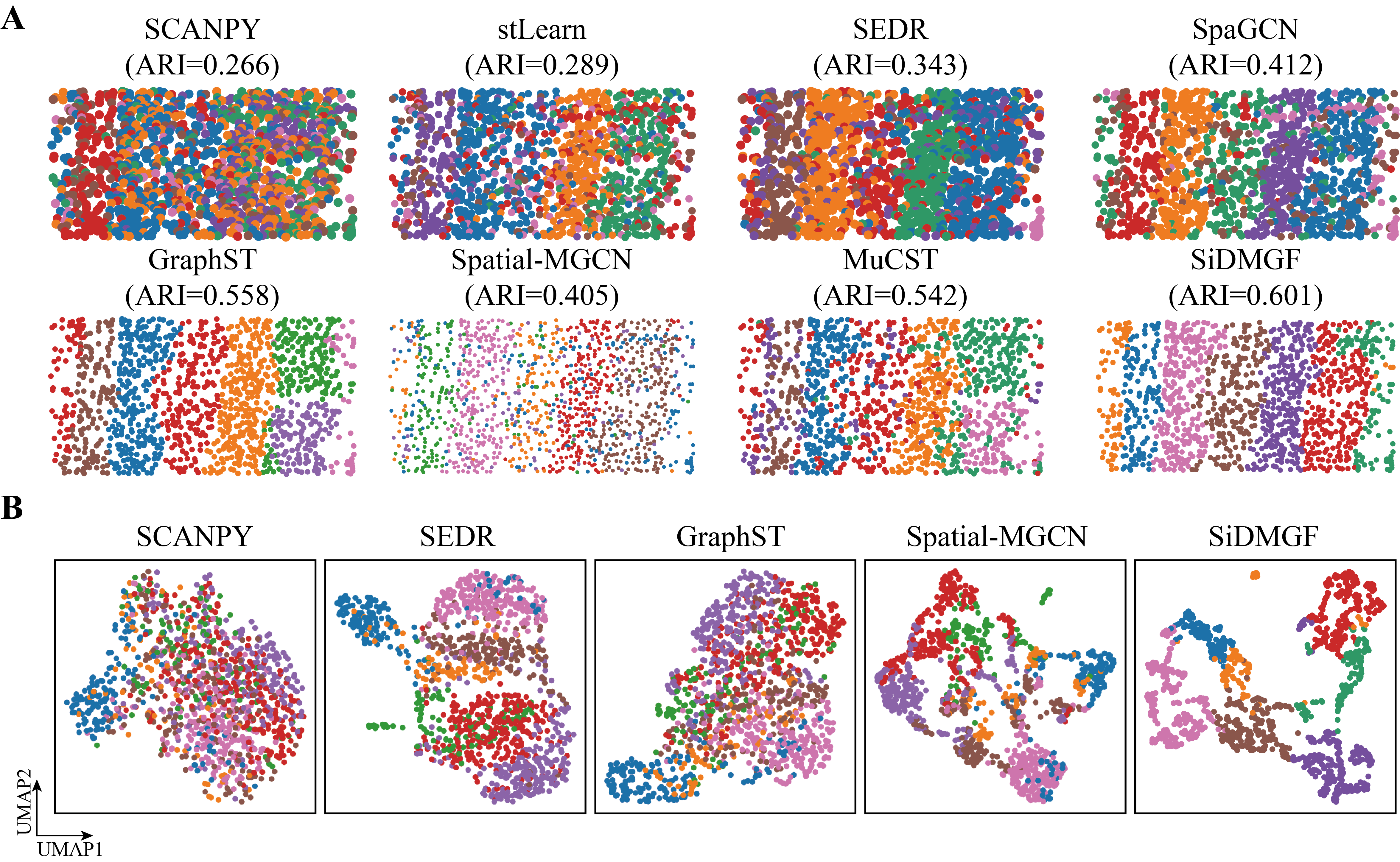


**Fig. S8** **(A)** Visualization of spatial domains identified by SCANPY, stLearn, SEDR, SpaGCN, GraphST, Spatial-MGCN, MuCST, and SiDMGF on STARmap data. **(B)** UMAP plots of cell embeddings learned by SCANPY, SEDR, GraphST, Spatial-MGCN, and SiDMGF, where spots are colored by the identified spatial domains.


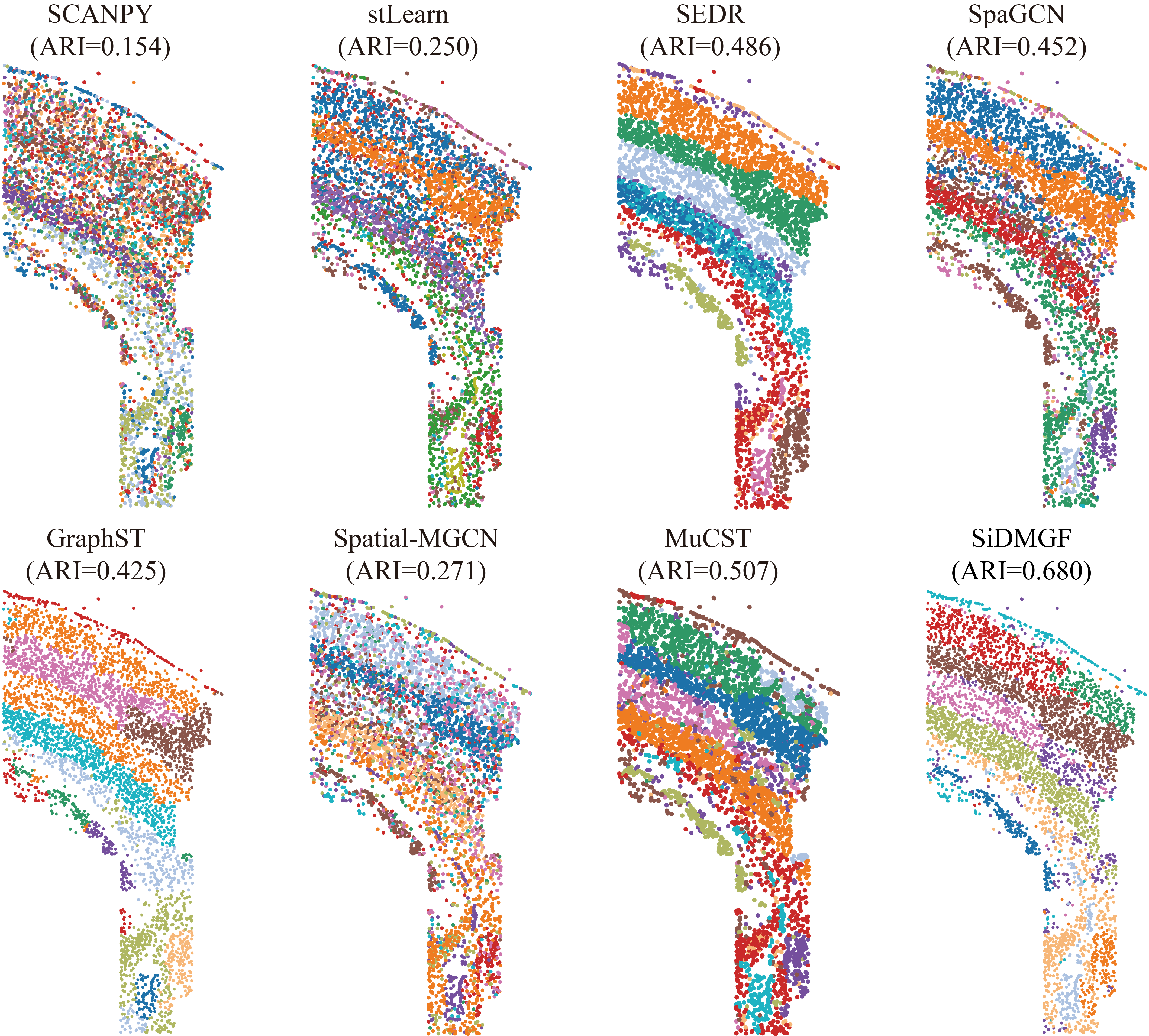


**Fig. S9** Visualization of spatial domains identified by different algorithms on mouse cortex slice sequenced by osmFISH, where spots are colored by the identified spatial domains.


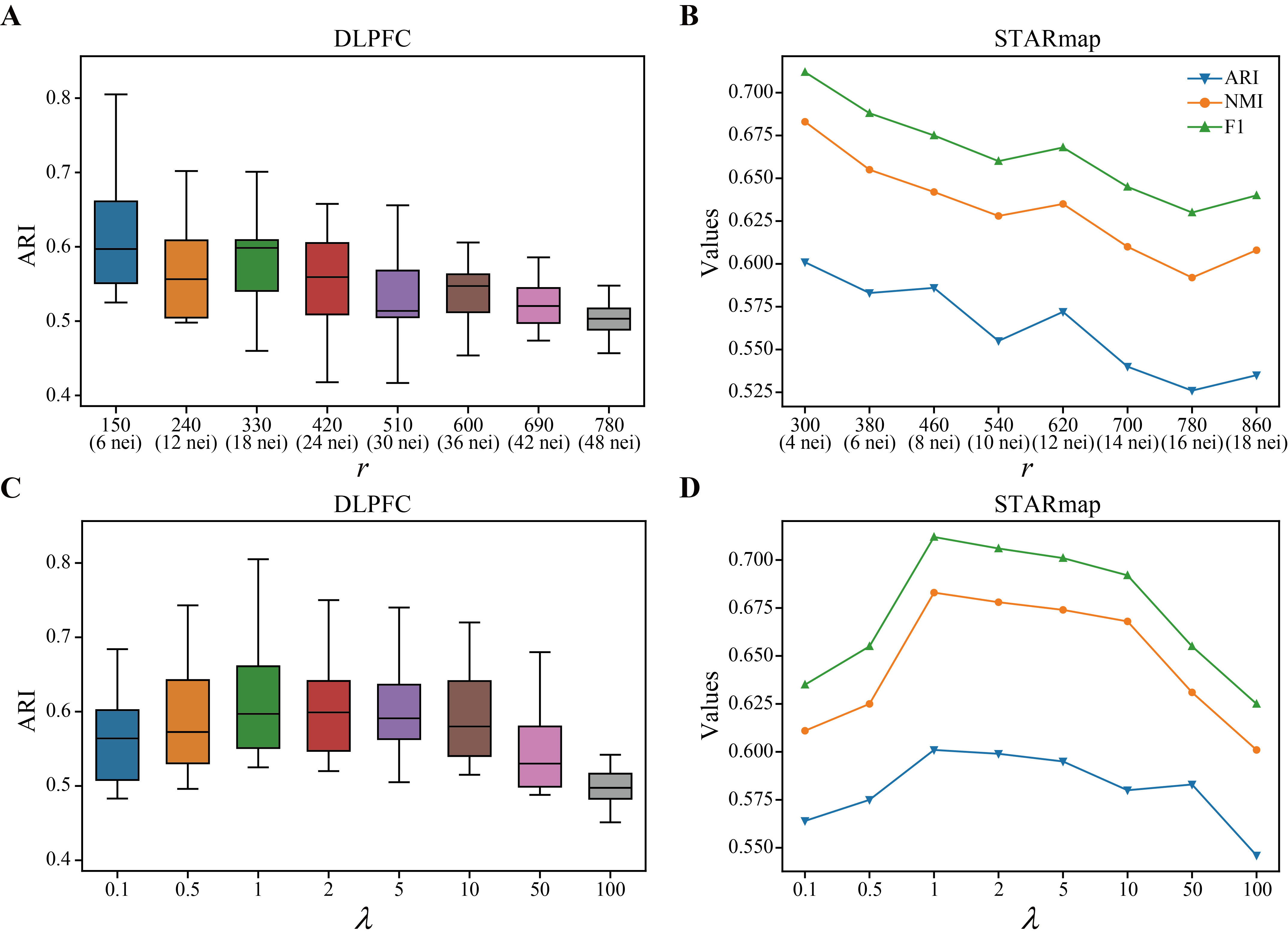


**Fig. S10** Parameter analysis of GEASO. **(A)** Analysis of the spatial radius r on human dorsolateral prefrontal cortex (DLPFC) sequenced using 10$\times$ Visium. **(B)** Analysis of the trade-off parameter $\lambda$ on human DLPFC dataset. **(C)** Analysis of the spatial radius $r$ on mouse cortex layer dataset sequenced using STARmap. **(D)** Analysis of the trade-off parameter $\lambda$ on STARmap dataset.
